# Inflammation and genetic factors in stroke pathogenesis

**DOI:** 10.20517/2347-8659.2017.22

**Published:** 2017-12-08

**Authors:** Hua Su

**Affiliations:** Center for Cerebrovascular Research, Department of Anesthesia and Perioperative Care, University of California, San Francisco, CA 94143, USA

Stroke is the leading cause of serious, long-term disability in the United States. Each year, approximately 795,000 people suffer a stroke. About 600,000 of these are first attacks, and 185,000 are recurrent attacks. Nearly three-quarters of all strokes occur in people over the age of 65 (www.strokecenter.org/patients/about-stroke/stroke-statistics/). According to the American Stroke Association, stroke is the 5th leading cause of death in the United States, killing 130,000 every year. The prevalence of stroke and its cost will undoubtedly rise as the aging population increases. In addition, stroke incidence and mortality are increasing in less developed countries in which the lifestyles and population restructuring are rapidly changing.

After ischemic stroke, severe disturbance of the blood supply to brain tissue deprives oxygen supply to brain tissue causing neuronal death. Pathophysiological events occur after ischemic stroke include ionic imbalance, neuroinflammation, excitotoxicity, activity of microglia. All of these contribute to neuronal death. Ischemia induces a significant increase in microvascular density, a sign of angiogenesis, in the penumbra of the cerebral infarct. The degree of increased vessel-density in the ischemic penumbra is positively correlated with the survival rate of stroke patients. In addition, increased angiogenesis was associated with an improved functional outcome after ischemic stroke in both animal models and in stroke patients. However, despite considerable research efforts, the exact mechanisms for stroke injury have not been fully understood. None of neuroprotection and angiogenesis strategies has shown therapeutic benefit in clinical setting. Administration of intravenous recombinant tissue plasminogen activator (rtPA) is still the only effective treatment. The therapeutic window of rtPA is limited within 4.5 h after the onset of ischemic stroke^[1,2]^. Endovascular treatment has been shown to improve functional outcome in 5 randomized clinical trials on selected patients with acute ischemic stroke. Thrombectomy with stent retrievers is now recommended as the standard of care for acute ischemic strokes with a proximal large vessel occlusion in the anterior circulation. However, the therapeutic window for this treatment is also limited^[1]^.

Cerebral ischemia induces a cascade of inflammatory and immune reactions that encompass genomic as well as molecular and cellular events. Immune response has been shown to play a major role in ischemic stroke progression. The extent of neuronal damage seems to correlate with the degree of innate immune activity. For example, bone fracture induced systemic inflammation exacerbates ischemic stroke injury in mice^[3,4]^ and reduction of inflammation through activation of a-7 nicotinic acetylcholine receptor reduces ischemic brain injury^[5]^. However, in recently years, many studies showed that neuroinflammation appear to be double-edged swords in the battle for neurological recovery. For example, microglia/macrophage activation fosters brain recovery by clearing cell debris, which leads to resolving local inflammation. Activated microglia/macrophage also produce a plethora of trophic factors that promote tissue repair^[6]^. Recent studies show that blocking CCR2 macrophage impairs functional recovery of stroke victims^[7]^. However, the kinetics of macrophage/microglia polarization switch is different among different models, such as reperfusion *vs*. permanent occlusion^[7,8]^. Therefore, the contradictory functions of microglia/macrophage might reflect their acquisition of distinct phenotypes in response to different microenvironmental cues^[6]^.

This issue includes a review and a mini review what discussed the role of inflammation in brain injury. Marcet *et al*.^[9]^ discussed the impacts of inflammation in ischemic stroke and traumatic brain injury in their review. They indicated that although the brain damage induced by the initial trauma is most likely unsalvageable, the secondary immunologic deterioration of neural tissue gives many opportunities for therapeutic strategists. This review highlighted the cell death mechanisms associated with injury or center nervous system (CNS) with special emphasis on inflammation. They discussed the sources of inflammation, and introduced the role of the spleen in the systemic response to inflammation after CNS injury. In the mini-review, Liu *et al*.^[10]^ overviewed the role of microglia in neuroinflammatory after ischemic stroke. In addition, a clinical study presented by Lu *et al*.^[11]^ in this issue demonstrated a correlation of cerebral microbleed and the level of inflammatory markers in blood.

In addition to inflammatory, other such as genetic variations influence stroke occurrence and recovery. A review in this issue by Zhu *et al*.^[12]^ discussed the roles of *endoglin* gene in cerebral vascular diseases. Endoglin (ENG) is a transforming growth factor beta associated receptor and is required for both vasculogenesis and angiogenesis. In human, ENG haploinsufficiency is associated with type 1 hereditary hemorrhagic telangiectasia (HHT), also known as Osler-Rendu-Weber Syndrome, which is an autosomal dominant disease. HHT patients have a higher prevalence of telangiectases in mucocutaneous membrane and arteriovenous malformation (AVM) in multiple organs. AVMs are abnormal vessels shunting blood directly from arteries to veins^[13]^. AVM has abnormal vessel wall structure, which is likely to rupture. Rupture of brain AVM can result in life-threatening intra-cranial hemorrhage and hemorrhagic stroke^[13]^. Alternatively, pulmonary AVMs in HHT type 1 patients are associated with a higher incidence of paradoxical embolism in the cerebral circulation causing ischemic brain injury than general population.

ENG is required for the differentiation and sprouting of endothelial tubes, which are important processes of angiogenesis. ENG is also an important mediator of endothelial-mesenchymal communication during angiogenesis. *Eng* deficient mouse embryos show impaired recruitment of vascular smooth muscle cells and pericytes to newly form vascular network. Zhu *et al*.^[12]^ discussed, in this issue, the roles of ENG in ischemic stroke and indicated that ENG expression might be a potential biomarker for vasospasm after subarachnoid hemorrhage and cerebrovascular stenosis. Experimental or therapeutic modulating of ENG expression could be useful in generation of animal models for study disease pathogenesis and for development of novel treatments for multiple cerebrovascular diseases.

In summary, we have tremendously expanded our working knowledge of how vascular remodeling in the brain occurs and identified many the key cellular and molecular events underlying this process in recently. In this issue, we have assembled a collection of articles from renowned experts in the field of brain injury and neuroinflammation, and attempted to lift some of the veil on the pathophysiology of stroke.
